# Bacopaside I ameliorates cognitive impairment in APP/PS1 mice via immune-mediated clearance of β-amyloid

**DOI:** 10.18632/aging.100913

**Published:** 2016-03-02

**Authors:** Yuanyuan Li, Xing Yuan, Yunheng Shen, Jing Zhao, Rongcai Yue, Fang Liu, Weiwei He, Rui Wang, Lei Shan, Weidong Zhang

**Affiliations:** ^1^ School of Pharmacy, Second Military Medical University, Shanghai 200433, P.R. China; ^2^ Department of Mathematics, Logistical Engineering University, Chongqing 401311, China; ^3^ School of Pharmacy, East China University of Science and Technology, Shanghai 200237, P.R. China; ^4^ Shanghai Institute of Pharmaceutical Industry, Shanghai 200040, P.R. China

**Keywords:** Bacopaside I, Alzheimer's disease, β-amyloid, immune, phagocytosis, APP/PS1mice

## Abstract

Standardized extracts of Bacopa monniera (BME) have been shown to exert a neuroprotective effect against mental diseases, such as depression, anxiety and Alzheimer's disease (AD), in chronic administration studies. However, its mechanism of action has remained unclear. In this study, we evaluated the therapeutic effect of Bacopaside I (BS-I), a major triterpenoid saponin of BME, on the cognitive impairment and neuropathology in APP/PS1 transgenic mice and explored the possible mechanism from a biological systems perspective. We found that BS-I treatment significantly ameliorated learning deficits, improved long-term spatial memory, and reduced plaque load in APP/PS1 mice. We constructed BS-I's therapeutic effect network by mapping the nodes onto the protein-protein interaction (PPI) network constructed according to their functional categories based on genomic and proteomic data. Because many of the top enrichment categories related to the processes of the immune system and phagocytosis were detected, we proposed that BS-I promotes amyloid clearance via the induction of a suitable degree of innate immune stimulation and phagocytosis. Our research may help to clarify the neuroprotective effect of BME and indicated that natural saponins target the immune system, which may offer new research avenues to discover novel treatments for AD.

## INTRODUCTION

Alzheimer's disease, which is clinically characterized by extracellular amyloid plaques in the extracellular region and neurofibrillary tangles within cells, is one of the most common neurodegenerative diseases in the world [1]. β -amyloid (Aβ), an amyloid precursor protein (APP) that is abnormally cleaved by the β-site APP cleavage enzyme 1 (BACE1) and γ-secretase, significantly contributes to AD pathology; the accumulation of this peptide in the hippocampus and cortex causes neuronal cell dysfunction and cognitive deficits in AD patients [2]. Immunotherapy that targets Aβ is considered the most promising approach to treat AD, because it can potentially reduce the production, aggregation and deposition of Aβ [3, 4]. However, the severe adverse reactions of present immunotherapy approaches limit their clinical application [5-7]. The advantages of botanical drugs for the treatment of AD suggest that phytotherapy holds significant promise in the treatment of AD [8, 9].

Bacopa monniera (L.) Wettst. (Brahmi), a member of Scrophulariaceae family, has been used in Indian Ayurvedic medicine to enhance memory and intelligence. Standard B. monniera extract (BME) has been used to treat several types of dementia in India and America and has been shown to exert many clinical benefits [10, 11]. Pseudojujubogenin and jujubogenin have been found to be responsible for the neuroprotective and cognitive-enhancing effect of the standard extract [12-14]. Bacopaside I (BS-I), one of the triterpenoid saponins of BME, could be a potential phytotherapeutic drug for the treatment of AD. We have reported that BS-I ameliorates depression and ischemic brain injury [15, 16]. However, the in vivo efficacy of BS-I for the treatment of AD has not yet been studied. In this study, we report for the first time that the chronic administration of BS-I reduces Aβ deposits and ameliorates cognitive impairments in a well-established strain of APP/PS1 transgenic mice. We then used systems biology methods to determine whether this triterpenoid saponin ameliorates cognitive impairment via the immune-mediated clearance of Aβ.

## RESULTS

### BS-I treatment effectively reverses learning and memory deficits in APP/PS1 mice

**Figure 1:**
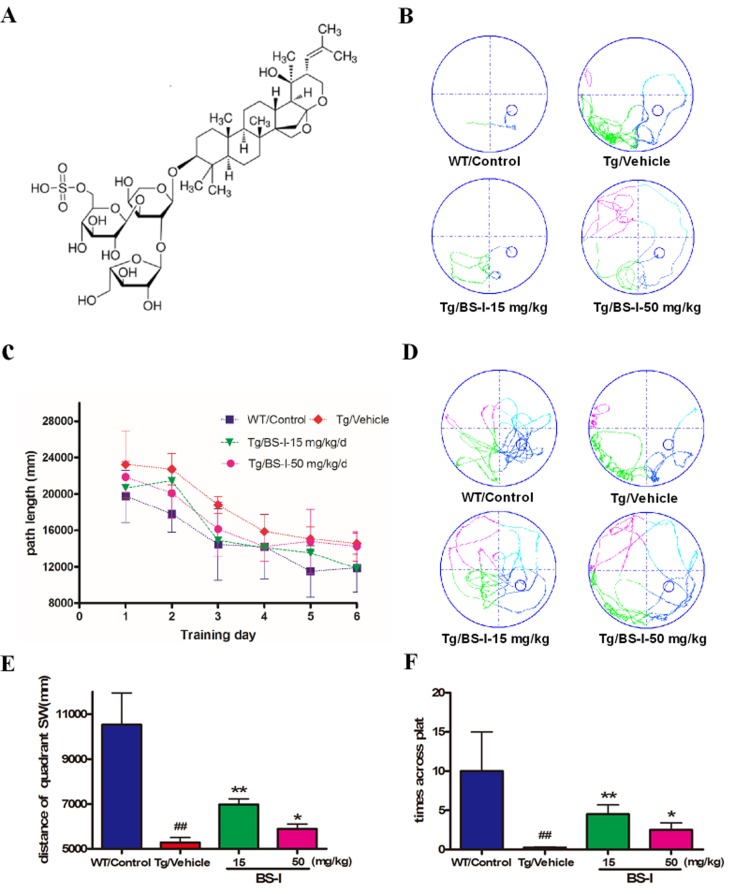
The chemical structure of BS-I (**A**) and Morris water maze test for BS-I and vehicle-treated APP/PS1 mice (WT controlled) (**B** to **F**). All mice were trained for 6 days (4 trials per day) to learn the location of a hidden platform in the MWM (**B** and **C**). Each point represents the mean length values of 4 trials per day. The swim path distance for the animals in the BS-I-treated groups were compared with those of vehicle-treated animals. Each mouse received a 70-second probe test of spatial memory retention on the 7th day (for 4 trials). In the probe trials, the BS-I-treated APP/PS1 mice swam longer in the target quadrant than the vehicle-treated APP/PS1 mice (**D**, **E** and **F**). APP/PS1/Vehicle group (Tg/Vehicle) compared with the wild-type control group (WT/Control), * p < 0.05, ** p < 0.01; BS-I-treated groups compared with Tg/Vehicle group, ## p < 0.01. Error bars denote mean and the standard error of the mean (SEM), n = 9.

Both Tg and WT mice were trained for 6 days and subjected to 4 trials each day in the MWM with a hidden platform to learn the position of the platform. The average distance to the platform for each trial was analyzed to determine the genotype and treatment differences in spatial learning. Vehicle-treated APP/PS1 mice swam significantly longer distances than WT-control group mice, from the 1st to the 6th day (p < 0.05) (Fig. 1B and 1C). The observed impairment in the spatial memory of APP/PS1 mice is consistent with that reported in a previous study [17]. Treatment with low and high doses of BS-I reduced the path distance in APP/PS1 mice compared with placebo-treated APP/PS1 mice (Fig. 1B and 1C). From days 3-4, APP/PS1 mice treated with 15 and 50 mg/kg per day of BS-I showed a reduction in swimming distance compared with vehicle control group mice, reaching values similar to those of the WT-control group (Fig. 1 B and 1C).

The MWM behavior testing confirmed that both doses of BS-I (15 and 50 mg/kg per day) not only significantly enhanced learning skills during the hidden-platform learning trial but also significantly enhanced spatial memory retention during the probe trial. We recorded the times the mice passed through the probe after removing the platform. Vehicle-treated-APP/PS1 mice crossed the target quadrant less frequently (p < 0.01) and swam a shorter path length than vehicle-treated WT mice (Fig. 1E and 1F). Compared with vehicle-treated APP/PS1 mice, the BS-I treated mice passed through the probe more often during the 90-s trial, especially the 15 mg/kg treated group (p < 0.01) (Fig. 1F). These results demonstrate that regular treatment with BS-I ameliorated spatial memory impairment in vehicle-treated APP/PS1 mice.

### BS-I mitigates Aβ level in APP/PS1 mice

**Figure 2:**
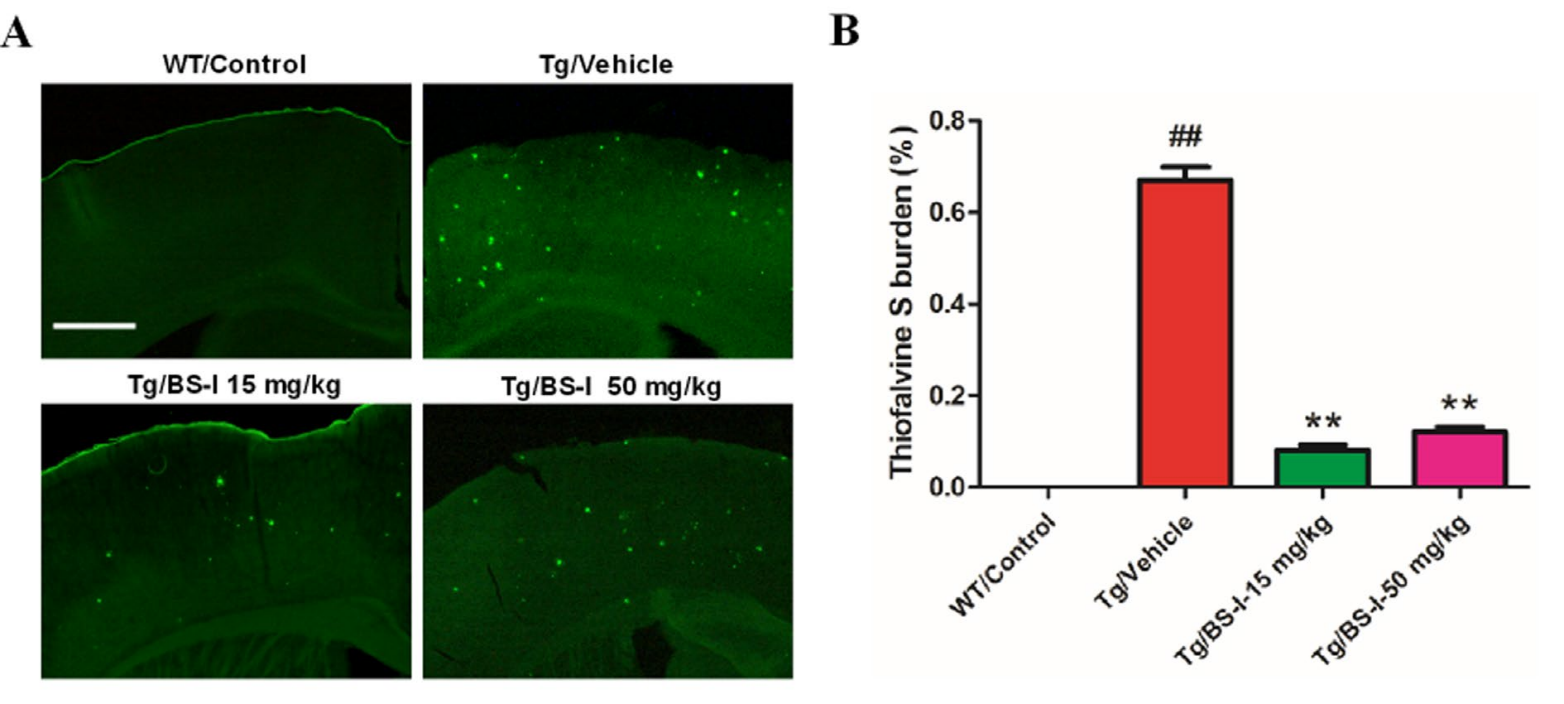
BS-I reduced amyloid plaque formation in APP/PS1 mice. (**A**) Images of ThioS-stained amyloid plaques in the hippocampus. (**B**) Statistics of ThioS-stained amyloid plaques in the hippocampus. APP/PS1/Vehicle group (Tg/Vehicle) compared with the wild-type control group (WT/**C**ontrol), ** p < 0.01; BS-I treated groups compared with Tg/Vehicle group, ## p < 0.01. Error bars denote mean standard error of the mean (SEM), n = 3. Scale bar, 100 μM.

The observation that BS-I treatment could alleviate learning and memory deficits led us to further examine possible correlations with the Aβ plaque deposition of BS-I in APP/PS1 mice. ThioS staining shows the intensive deposition of senile plaques in APP/PS1 mice, while fluorescent highlights are absent in the wild-type mouse brain. Consistent with the suggestion that BS-I treatment reduces the accumulation of Aβ peptides in the brain, we detected a remarkable reduction in ThioS-positive fibrillar Aβ in the brains of the APP/PS1 mice treated with 15 mg/kg BS-I per day (p < 0.01) compared with age-matched untreated control APP/PS1 mice (Fig. 2A and 2B). The digital images from the cortex and hippocampus were captured and analyzed with the ImageJ software (National Institutes of Health, Bethesda, MD, USA).

### Proteomics results and validation of differentially abundant proteins using Western blot

**Figure 3:**
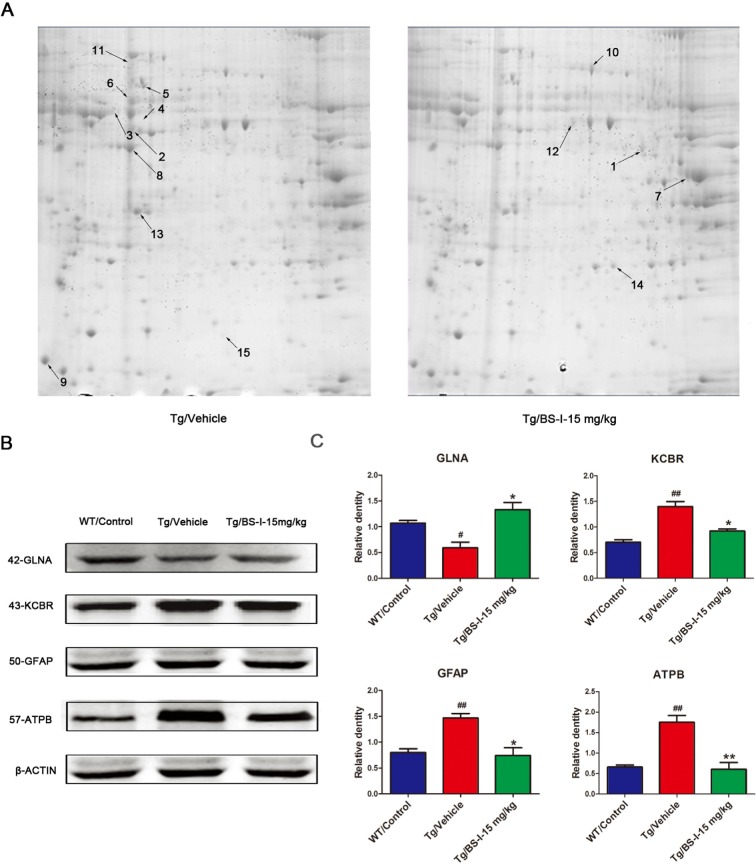
2-DE images and the validation of differentially abundant protein by western blot. The differentially expressed proteins in this study were defined by the gray values of the protein spots. Nine proteins were down-regulated (shown on the Tg/Vehicle gel) and 5 were up-regulated (shown on the Tg/BS-I-15 mg/kg gel) (**A**). To validate the proteomic results, we used a western blot to confirm 4 of the total 14 differentially expressed proteins (**B**). Tg/Vehicle group (M) compared with the wild-type control group (WT), * p < 0.05, ** p < 0.01; Low dose BS-I (15 mg/kg) treated group (L) compared with Tg/Vehicle group, # p < 0.05, ## p < 0.01 (**C**). Error bars denote mean standard error of the mean (SEM), n = 3.

We further carried out a comparative proteomic analysis using matrix-assisted laser desorption/ionization-time of flight (MALDI-TOF) MS/MS of BS-I treated and vehicle treated mouse brains. We found that 5 proteins, glutamine synthetase (GLNA), fructose-bisphosphate aldolase isoform 2 (ALDOA), alpha-enolase (ENOA), tubulin beta-2A chain (TBB2A) and triosephosphate isomerase (TPIS), were up-regulated (p < 0.05 vs. vehicle treated) and 9 proteins, glial fibrillary acidic protein (GFAP), ATP synthase subunit beta (ATPB), creatine kinase B-type (KCRB), alpha-internexin (ANIX), tubulin alpha-1C chain (TUBA1C), cytoplasmic beta-actin (ACTB), beta-synuclein (SYUB), heat shock protein 8 (SHP7C) and ATP synthase subunit alpha (ATPA), were down-regulated (p < 0.05 vs. vehicle treated) in BS-I-treated brains (Fig. 3A). Protein identifications based on MALDI-TOF MS/MS are listed in Table 1. We validated 4 proteins from all differentially abundant proteins using western blot: GLNA, ATPB, GFAP and KCRB (Fig. 3B and 3C).

**Table 1:** Differentially expressed proteins between the BS-I-treated APP/PS1 and vehicle-treated mice

Spot no.	Protein name	MW (kDa)	NCBI Accession no.	Ration (BS-I treated/Vehicle)
1	Glutamine synthetase	42.092	gi|145559476	2.25
2	glial fibrillary acidic protein	49.265	gi|73769823	0.51
3	ATP synthase subunit beta	56.265	gi|20455479	0.4
4	Creatine kinase B-type	42.686	gi|417208	0.27
5	alpha-internexin	55.349	gi|148539957	0.26
6	Tubulin alpha-1C chain	49.877	gi|55977482	0.20
7	Fructose-bisphosphate aldolase isoform 2	39.331	gi|113607	1.95
8	cytoplasmic beta-actin	39.161	gi|387083	0.35
9	Beta-synuclein	14.043	gi|81879780	only in M
10	Heat shock protein 8	70.828	gi|42542422	0.39
11	Alpha-enolase	47.111	gi|13637776	3.13
12	Tubulin beta-2A chain	49.875	gi|81885934	4.00
13	Triosephosphate isomerase	26.696	gi|2851390	1.37
14	ATP synthase subunit alpha	59.716	gi|416677	0.66

### Pathways enriched with AD-associated genes and proteins

**Figure 4:**
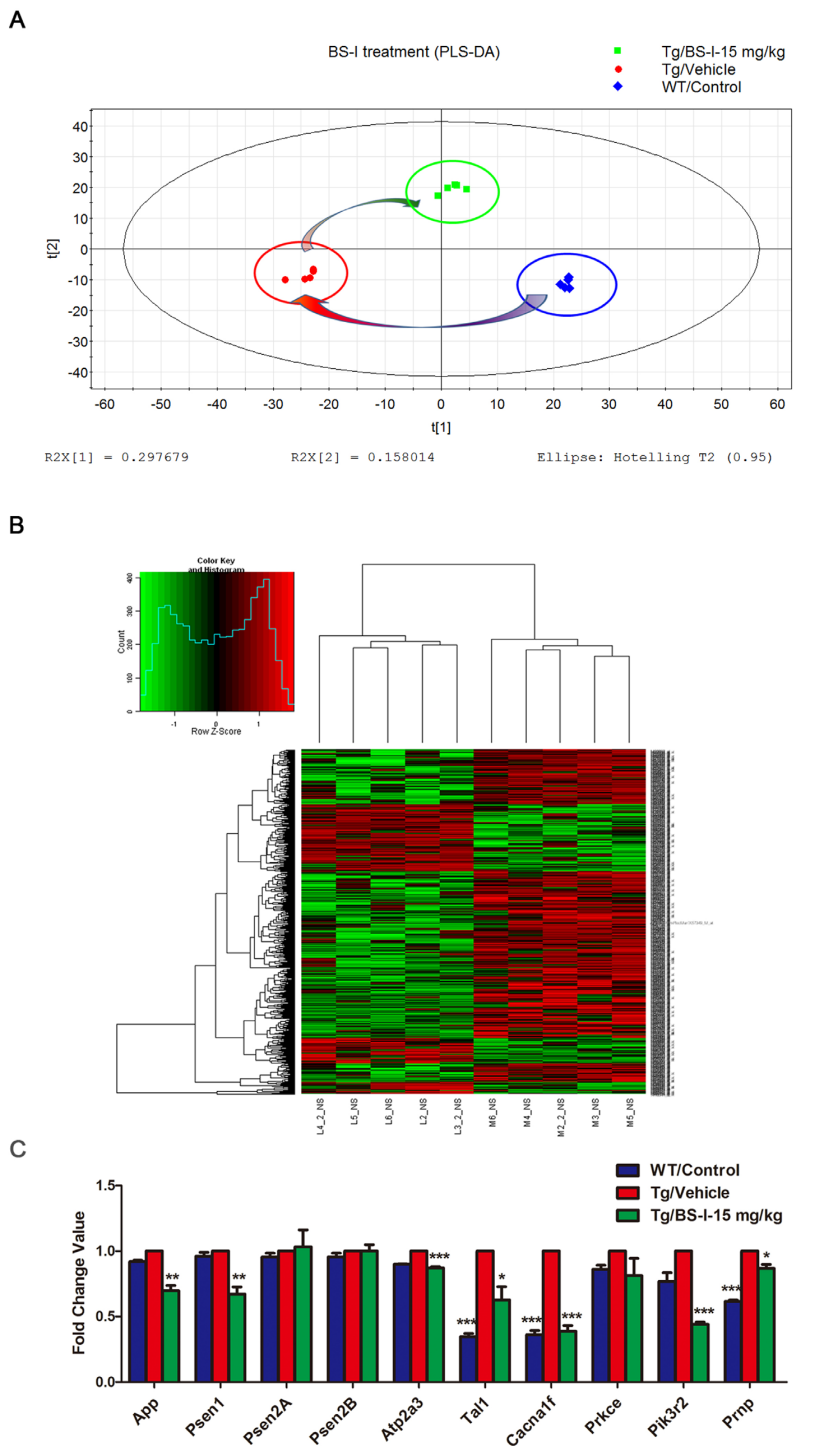
PLSDA analysis of all 45101 probes of three differently treated groups. (**A**). A heat map of all 568 differentially expressed genes between BS-I treated and vehicle-treated groups APP/PS1 mouse brains (**B**). The mRNA expression patterns of 10 selected genes in the brain of BS-I-treated and vehicle treated APP/PS1 mice compared with wild-type mice assessed by real-time PCR agreed with the cDNA microarray results (**C**). The data are presented as the standard error of the mean (SEM); * p < 0.05, ** p < 0.01, *** p < 0.001.

The PLSDA map of all 45101 probes and the heat map of all differentially expressed genes indicates that the transgenic mice differ greatly from the wild-type mice and that the BS-I treatment had a significant effect on the AD mice (Fig. 4A and 4B).

We found 568 differentially expressed (397 down-regulated and 171 up-regulated) probes in the BS-I treated group (L) compared with the vehicle-treated APP/PS1 group (M) and 823 differentially expressed probes (318 down-regulated and 505 up-regulated) in the APP/PS1 group (M) compared with the wild-type control group (L) with p-values < 0.05 and abs (fold change value) >2. A pathway enrichment analysis was conducted to determine pathways that were significantly impacted by BS-I treatment, and the p-values were computed for all pathways that included AD-associated proteins. The computations generated 15 pathways that were mostly involved in BS-I -mediated anti-AD effects: phagosome, endocytosis, neuroactive ligand-receptor interaction, Alzheimer's disease, calcium signaling pathway, MAPK signaling pathway, natural killer cell mediated cytotoxicity, Fc gamma R-mediated phagocytosis, antigen processing and presentation, hematopoietic cell lineage, chemokine signaling pathway, T cell receptor, Toll-like receptor, neurotrophin signaling pathways and apoptosis pathways (p < 0.05, Table 2). We validated 10 genes from the gene chips (App, Psen1, Psen2A, Psen2B, Atp2a, Tal1, Cacna1f, Prkce, Pik3r2 and Prnp) in the brains of BS-I-treated and vehicle treated APP/PS1 mice. The mRNA expression patterns of the 10 genes assessed using real-time PCR were consistent with the cDNA microarray results (Fig. 4C).

**Table 2:** Top 15 significantly enriched GO terms for differentially expressed genes between the BS-I-treated and vehicle-treated APP/PS1 mice

Pathway Class	Pathway Name	Pathway gene	Mapped pathway gene	p-value
Transport and Catabolism	Phagosome	136	12	0.0001
Transport and Catabolism	Endocytosis	240	10	0
Signaling Molecules and Interaction	Neuroactive ligand-receptor interaction	331	10	0.0004
Neurodegenerative Diseases	Alzheimer's disease	284	9	0.0006
Signal Transduction	Calcium signaling pathway	204	9	0.0001
Signal Transduction	MAPK signaling pathway	284	8	0.0024
Immune System	Natural killer cell mediated cytotoxicity	161	6	0.0022
Immune System	T cell receptor signaling pathway	132	6	0.0008
Immune System	Fc gamma R-mediated phagocytosis	105	6	0.0003
Immune System	Antigen processing and presentation	104	6	0.0002
Immune System	Toll-like receptor signaling pathway	104	6	0.0002
Immune System	Hematopoietic cell lineage	86	5	0.0008
Immune System	Chemokine signaling pathway	203	5	0.025
Nervous System	Neurotrophin signaling pathway	144	24	0
Cell growth and death	Apoptosis	95	3	0.0425

### The therapeutic effect network of BS-I effect on AD

**Figure 5:**
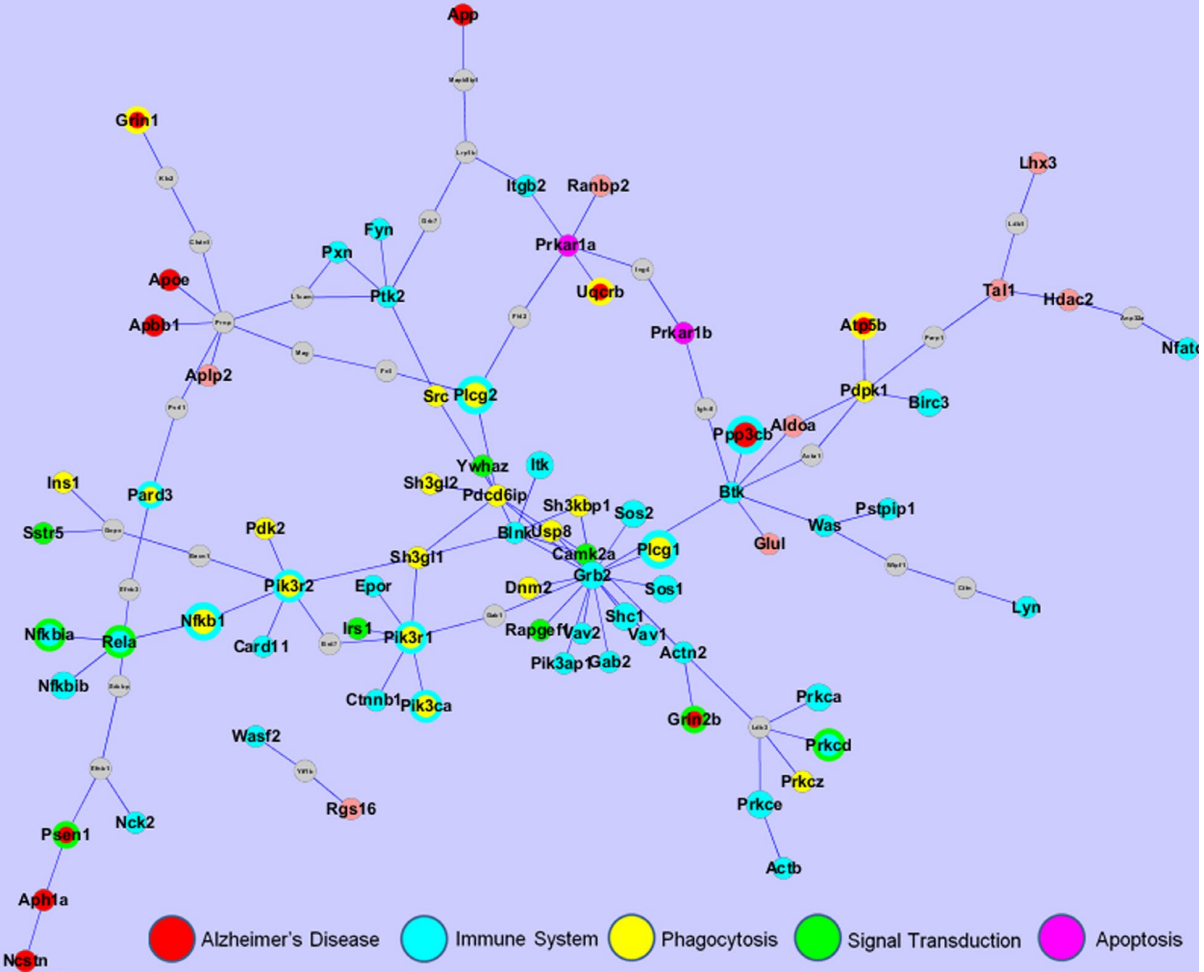
The PPI network of the effect of BS-I on AD. The network was constructed by mapping the most enriched functional genes to the PPI network constructed by the differentially expressed genes identified by the DNA microarray and differentially expressed proteins identified by the 2-DE. Immune genes are colored in blue and phagocytosis genes are colored in yellow. Multi-functional genes are identified by multiple colors. The size of a node depends on the number of pathways the gene is involved in. Grey nodes are non-target proteins that link the targets.

Recent advances in experimental biology have enabled the production of vast amounts of protein-protein interaction (PPI) data. Thus, the use of PPI data to function ally annotate proteins has been extensively studied. Therapy-associated networks are expected to aid the understanding of the effect of BS-I on AD as well as the identification of potential target sets of BS-I on AD or other neurosystem diseases. In this work, we integrated the 568 differentially expressed probes and 14 differentially expressed proteins of the BS-I-treated group compared with the vehicle-treated group to determine the specific PPI network of the BS-I effect on AD, which included 105 nodes and 113 edges (Fig. 5).

Each differentially expressed gene was allowed to interact with other target genes via no more than one non-target gene. The nodes are colored according to their biofunctional categories. Most of the genes in the PPI network are involved in five functional categories: the immune system, phagocytosis, the existing AD pathway, signal transduction and apoptosis.

## DISCUSSION

The pathogenesis of Alzheimer's disease, which currently lacks a curative treatment, is associated with the accumulation of amyloid β (Aβ) in the brain. Many recently published studies have found that some natural small molecules can significantly clear Aβ from the brain. BM is classified in classical Indian Ayurveda medicine system as a plant-derived drug and is used as a nervine tonic to ameliorate mental health and improve memory and intellect. Earlier experimental and clinical studies have demonstrated the memory-promoting and cognitive-enhancing action of the plant extract. Although BME is a well-known neuroprotective agent[18], the actual therapeutic role and molecular mechanism of its major active component, BS-I, has not yet been evaluated in AD pathology. The data from our study represent the first evidence to support that the prolonged administration of BS-I can ameliorate the age-related cognitive impairments and Aβ accumulation observed in APP/PS1 mice[19]. Both low (15 mg/kg) and high (50 mg/kg) doses of BS-I significantly reduced the cognitive impairment of AD mice based on a conventional reference memory MWM task and a memory retention task (probe trial) (Fig. 1C, E and F). As reported, senile plaques that consist of Aβ are a characteristic feature of AD and a primary criterion in the neuropathological-histological verification of AD[20]. Therefore, we evaluated the ability of BS-I to reduce amyloid plaque formation in mice. Notably, BS-I-induced decreases in memory deficits were accompanied by a remarkable reduction in the amyloid burden in the brains of transgenic mice (Fig. 2A and 2C). In this study, we found that the low dose of BS-I was more potent than the high dose in reducing Aβ pathology. A similar pattern has been reported for the Aβ-reducing effects of memantine [21] and berberine [22].

The ability of natural small molecules to modulate complex diseases is usually due to the regulation of multiple genes [23]. Therefore, a systems approach based on a network may be conducive to the identification of candidate genes related to the complex modulation of AD by BS-I. In this paper, we studied the network of therapeutic effects of BS-I by integrating proteomic and microarray data obtained from BS-I-treated APP/PS1 mouse brains to determine the candidate genes related to BS-I effects in the context of APP/PS1 mouse protein interactions. Based on the top-ranked differentially expressed genes identified by the microarray chips and the differentially expressed proteins identified by the 2-DE (Table 1), we constructed a specific BS-I effect PPI network, identified potential pathways associated with BS-I effects, and finally sketched an overview of the biological processes involved in the therapeutic effect of BS-I in AD. BS-I modulated a variety of signaling pathways in AD. Among these pathways, 15 pathways were found to be of major importance in the present study (Table 2). We speculate that these pathways likely mediate most of the effects of BS-I treatment. The PPI network of the differentially expressed genes and proteins were then constructed to explore the relationships between these genes. Our network analysis indicates that the modulation of a combination of targets likely mediated the therapeutic effect of BS-I. Target proteins that crosstalk between multiple pathways are of particular interest, such as Grin1, Uqcrb, Atp5b, Plcg2, Ppp3cb, Plcg1, Nfkbia, Rela, Nfkb1, Grin2b, Pik3r1, Pik3r2, Pik3ca, Psen1 and Prkcd. This crosstalk allows BS-I to intervene in multiple pathways involved in AD via a limited number of targets. Furthermore, the multiple-target mechanism identified in our study could be valuable for designing new multi-target drugs for AD. The down regulation of ATPB and ATPA in the BS-I treated group indicated that BS-I could also reduce the oxidative stress level, which was supported by the improvement of improved enzyme activities and reduction of MDA in the mouse brain (Fig. S1).

In summary, we proposed that BS-I disturbs system of networks in the APP/PS1 mouse brain. Our data show that the biological processes most involved in BS-I regulation are part of the immune system, such as the regulation of natural killer cell-mediated cytotoxicity, Fc gamma R-mediated phagocytosis, antigen processing and presentation, hematopoietic cell lineage, chemokine signaling pathway, T cell receptor and Toll-like receptor pathway. The involvement of these pathways indicates, the immunomodulatory feature of BS-I. The immunomodulatory effect of BS-I in APP/PS1 mice indicates the therapeutic potential of this drug for AD, because neuro-inflammation is a critical risk factor for neurodegenerative disease, and the modulation of the immune system is widely accepted to be able to influence disease progression. Genome-wide association studies (GWAS) have identified several genetic risk loci for LOAD that seem to cluster in patterns that suggest immunity and endocytosis as important causal biological processes [24]. Many studies have reported that finely tuned innate immune activation is highly effective in the treatment of AD [25-29]. Therefore, BS-I seems to induce a suitable degree of innate immune stimulation that reduces the accumulation of Aβ and enhances spatial memory in APP/PS1 mice. Evidence of the influence of saponin on T cells and immunity supports our results [30]. In normal Aβ production, endocytosis brings the APP membrane protein into the early endosome, where Aβ is produced. Aβ is then recycled back to the plasma membrane[31]. Phagocytosis is a central process during remodeling, inflammation, and defense against infection in which cells take in relatively large particles. Microglia may exert neuroprotective effects by clearing Aβ via increased phagocytosis [25]. The microglia in the AD brain are poor phagocytes for deposited amyloid. This phenomenon has been suggested to be partly due to the inhibition of the phagocytic capacity of microglia by the chronic inflammatory environment [32]. We proposed that BS-I clears Aβ via the induction of an appropriate degree of innate immune-mediated phagocytosis. However, the mechanism by which BS-I regulates the body to reach an appropriate level of innate immunity is unclear and requires further research.

In conclusion, our study demonstrate that BS-I can reduce the Aβ level in the brains of AD mice, protect neurons from injury, improve the activity of anti-oxidative enzymes and ameliorate cognitive impairment in the APP/PS1 mouse model. We used genomic and proteomic data analysis, we propose the molecular network of BS-I in AD therapy, which may modulate key signaling pathways of the immune system and phagocytosis. To the best of our knowledge, this report is the first to describe the mechanism and molecular signaling network of BS-I using a systems biology strategy. Many reports have found that saponins have neuroprotective activity and proposed various hypotheses for the mechanism of this activity [33, 34]. Our research yielded a more credible hypothesis based on systemic data. Specifically, saponins may regulate the immune and inflammatory responses to play a neuroprotective role. Studies reporting the immunomodulatory and anti-inflammatory activity of saponins support our conclusion [30, 35, 36]. The results from our study will guide future studies of BS-I-like saponins as candidates for Aβ-based therapeutics to modify Aβ pathology in AD.

## MATERIALS AND METHODS

### Chemicals and reagents

BS-I (purity is 90.2%) was obtained and identified in our laboratory following previously described methods [37, 38]. For oral administration (via gavage), BS-I was diluted in 0.5% carboxymethylcellulose (CMC)-saline. The volume of oral administration was 0.1 mL/10 g body weight.

### Animal treatment and sample preparation

A group of 36 male mice consisting of 27 APP/PS1 mice and 9 age-matched wild type C57BL/6J mice was obtained from the Yikelihao Biotechnology Co. (Beijing, People's Republic of China). All mice were maintained on ad libitum food and water in a controlled environment (20–21°C; humidity, 50–60 %; light/dark cycle, 12 h/12 h). The mice were divided into different treatment groups (9 for each group) at random after being allowed to adapt to the local conditions for 2 weeks: WT mice (WT/Control group), APP/PS1 control mice (Tg/Vehicle), APP/PS1 mice with a low dose of BS-I treatment (APP/PS1 /BS-I-15 mg/kg per day) and APP/PS1 mice with a high dose of BS-I treatment (APP/PS1/BS-I-50 mg/kg per day). BS-I administration was initiated at 6 months of age and continued for 2 months until the mice were 8 months of age. The doses of BS-I were selected in accordance with our previous studies [15, 16]. The behavioral tests and neurochemical examinations were conducted 40 min after BS-I treatment. All experiments in this study were carried out according to the Guidelines for the Care and Use of Laboratory Animals of the Second Military Medicine University, Shanghai, China.

### Morris water maze

After 2 months of BS-I administration, the spatial memory of all 36 mice was assessed in Morris water maze (MWM) as described by Morris with few modifications [39]. The maze consisted of a white circular pool approximately 100 cm in diameter that was filled with opaque water (20±1°C). The pool was separated into four quadrants, NE, SE, SW and NW. A white platform was submerged in the SW quadrant of the pool 1 cm beneath the surface of the water to make it invisible to the mice while swimming. The mice underwent hidden platform training for 6 consecutive days (4 trials per day). During the hidden-platform test, one distal visual cue was used on the black screen in the middle of each quadrant around the pool. For each test, the mice were permitted to swim for a maximum time of 70 seconds to find the hidden platform, starting from release facing the pool wall in a randomly chosen quadrant. When successful, the mouse was allowed a 30-sec rest period on the platform. Mice that failed to reach the platform were physically placed on it and also allowed to rest for 30-seconds. Two trials were separated by a 30-minute interval on each testing day.

A probe trial was conducted on the 7th day to assess the memory retention of the mice. In this test, the platform was removed from the target quadrant and the mice were allowed to swim freely for 70 seconds to search for the hidden platform. During each test, the time and distance the mice spent in the SW quadrant were recorded to indicate the level of memory.

### Thioflavin S of A β levels

After the MWM experiment, three mice in each group were deeply anaesthetized with an intraperitoneal injection of sodium pentobarbital and perfused with 4% paraformaldehyde. The paraformaldehyde-fixed brain tissues were obtained immediately and embedded in paraffin. Ten-micrometer-thick coronal sections were stained for thioflavin S (ThioS) according to a previous approach [40]. The sections were stained in 1% ThioS for 5 min, differentiated in 70% alcohol for 5 min and mounted in glycerin jelly. One section from each mouse was used for plaque quantification with ThioS staining. The ThioS plaque burdens were counted throughout the entire hippocampus and cortex (3 mice per group) using the Image-Pro Plus software (Media Cybernetics).

### Two-dimensional electrophoresis (2-DE) and image analysis

Whole brain homogenate (0.1 g) from each group, i.e., the wild-type group, Tg group or Tg group with a low (15 mg/kg) BS-I treatment (3 per group), were mixed and homogenized in 9 volumes of tissue lysis buffer containing 9 M urea, 4% CHAPS, 65 mM DTT, 0.5% carrier ampholytes Bio-Lyte 3-10 (Bio-Rad) and protease inhibitors. The suspensions were sonicated for 40 s and centrifuged for 1 h at 12,000 rpm (4°C) to remove RNA, DNA and any other particulates. The supernatants contained all brain proteins that had solubilized in the sample buffer. The supernatant protein concentrations were measured using a Bradford Assay Kit (Bio-Rad). 2-DE was conducted according to the manufacturer's (Bio-Rad) protocol. For the first dimension, 1 mg of protein was applied to immobilized 17-cm, pH 3-10 nonlinear IPG strips (Bio-Rad). Rehydration was achieved at 50 V for 12 h. Isoelectric focusing (IEF), using a Protean i12 IEF System (Bio-Rad) was started at 250 V, and the voltage was gradually increased to 10,000 V and maintained constant until 80,000 Vh. The second dimension separation was performed on 10% SDS-PAGE gels (200 mm ×200 mm×1.0 mm) at a constant current of 30 mA/gel. The 2D gels were stained with Coomassie blue G-250 over night after electrophoresis[41], and the gels were scanned with an Image Scanner (UMAX 2100XL). Spot detection, matching and a quantitative intensity analysis were carried out using the PDQuest 8.0 software (Bio-Rad). The intergroup comparison of the samples between the Tg-Control group and BS-I-treated (15 mg/kg) group was carried out using three paired gels. Similar qualitative changes had to be detected in all three paired repeats to ensure that changes in the protein spots reflected actual differences in protein expression.

### MALDI-TOF-MS and database searching

Each sample was suspended in 0.7 μL of matrix solution, and the mixture was immediately spotted onto the MALDI target and allowed to dry and crystallize. The analyses were performed using a 4700 Proteomics Analyzer (TOF/TOF) (Applied Biosystems, Foster City, CA, USA). The proteins were identified using peptide mass finger printing (PMF) and tandem mass spectrometry (MS/MS) using the MASCOT program (version 1.9, Matrix Science, London, U.K.), and the data were compared to the Swiss-Prot database with the GPS Explorer software (Applied Biosystems); MAS COT protein scores (based on the combined MS and MS/MS spectra) greater than 56 were considered statistically significant (p < 0.05).

### Western blot analysis

Western blot analyses were carried out according to a previously described method [42]. Equal amounts of protein were subjected to 10% SDS polyacrylamide gel electrophoresis, transferred onto NC membranes at 220 mA for 1 hour, and then blocked with 5% fat-free milk in TRIS-buffered saline containing 0.1% Tween 20 at room temperature for 2 hours. The membrane was then incubated with the selected antibodies overnight at 4°C. The antibodies used for the western blots were β–actin (1:1000; CST, USA), GFAP (Rabbit Monoclonal Antibody, 1:1,000, Epitomics), creatine kinase B type(1:10,000 - 50,000, Epitomics), GLUL(1:1,000, Abcam) and anti-ATPB antibody(1:500, Abcam). The immunoreactive bands were visualized using Odessey and quantitatively analyzed using the Quantity One® 1-D Analysis Software (The Discovery Series™ Version 4.6.2).

### DNA microarray analysis and RNA analysis

At the end of the behavioral experiments, a necropsy was carried out on 5 mice from each group immediately after euthanasia. The removed brain was immediately stored in liquid nitrogen for subsequent genomic analysis. A 15 Affymetrix mouse 430 2.0 cDNA microarray (Bohao Biotech Ltd., Shanghai, China) was used in this study. The microarray contained 45101 cDNA probe sets. The total RNA from the test group was labelled with red-fluorescent Cy3-dUTP during reverse transcription, and the total RNA from the control group was labeled with blue-fluorescent Cy5-dUTP (both from Amersham Pharmacia Inc., Sweden). Reverse transcription was performed using Oligo-dT primers (Operon Inc., USA) and Super-Script II RNase H-Reverse Transcriptase (Invitrogen Inc., Carls-bad, CA) for 1 h at 42°C. After reverse transcription, probe cDNA was prepared as previously described [43].

Array hybridization and washing were performed using the GeneChip Hybridization, Wash and Stain Kit (Cat#900720, Affymetrix, Santa Clara, CA, US) and Fluidics Station 450 (Cat#00-0079, Affymetrix, Santa Clara, CA, US) following the manufacturer's instructions.

The images were converted to numerical information using ImaGene (Scan resolution 10 μm, PMT 100%), the data were standardized with the GeneSpring software, and the ratio is expressed as Cy3/Cy5. The discrepant genes were selected according to the following criteria: a ratio g 2 for up regulated genes and a ratio of 0.5 for downregulated genes. The final data are represented as a log-ratio (base2): log 2 (Cy3 / Cy5).

### Real-time PCR

The reliability of the cDNA microarray results was assessed using conventional reverse transcriptional polymerase chain reaction (RT-PCR) for the selected genes on both the Tg/vehicle-treated group and BS-I-treated (15 mg/kg) group brain tissues. The total RNA was prepared from brain tissue 40 min after MWM, and the cDNAs were prepared according to the manufacturer's instructions (Invitrogen, Carlsbad, USA); quantitative PCR was performed on the TP800 real-time PCR system (Takara Bio) using a standard SYBR Green PCR kit (Takara Bio). The relative expression of the measured genes in each sample was compared to GAPDH, and the significance was calculated using the average of the GAPDH-normalized 2-ΔΔCt value. PCR amplification primers see the Supplemental Material.

### Identification of pathways enriched with differentially expressed genes

The differentially expressed genes identified by our microarray experiments and the genes corresponding to differentially expressed proteins in the proteomic experiments were considered BS-I treatment-associated genes. These genes were mapped onto the mouse pathways in the KEGG database, and p-values were then used to determine whether a pathway was enriched with BS-I treatment-associated genes.

### Statistical analysis

The GraphPad Prism 5 software was used to perform the calculations and prepare graphical data (GraphPad Software, San Diego, CA, USA). The results are presented as the mean ± standard error of the mean (SEM). The behavioral data were analyzed with a two-way analysis of variance (ANOVA) for repeated measures.

## SUPPLEMENTARY DATA FIGURES



## Abbreviations

BS-I: Bacopaside I
AD: Alzheimer's disease
WT: wild type
Tg mice: transgenic mice
ThioS: thioflavin S
