# Erratum: Bacopaside I ameliorates cognitive impairment in APP/PS1 mice via immune-mediated clearance of β-amyloid

**DOI:** 10.18632/aging.100956

**Published:** 2016-04-22

**Authors:** Yuanyuan Li, Xing Yuan, Yunheng Shen, Jing Zhao, Rongcai Yue, Fang Liu, Weiwei He, Rui Wang, Lei Shan, Weidong Zhang

**Aging (Albany NY) 2015; 8(3)**: **521-538**.

PMCID: PMC 833143 PMID: 26946062

In this Article, the information on corresponding author is wrong: http://www.impactaging.com/papers/v8/n3/pdf/100913.pdf. The correct corresponding authors are provided here:

Correspondence: Weidong Zhang, PhD; Lei Shan, PhD; E-mail: wdzhangy@hotmail.com; shanleicn@126.com

