# Correction for: Bacopaside I ameliorates cognitive impairment in APP/PS1 mice via immune-mediated clearance of β-amyloid

**DOI:** 10.18632/aging.204531

**Published:** 2023-02-27

**Authors:** Yuanyuan Li, Xing Yuan, Yunheng Shen, Jing Zhao, Rongcai Yue, Fang Liu, Weiwei He, Rui Wang, Lei Shan, Weidong Zhang

**Affiliations:** 1 School of Pharmacy, Second Military Medical University, Shanghai 200433, P.R. China; 2 Department of Mathematics, Logistical Engineering University, Chongqing 401311, P.R. China; 3 School of Pharmacy, East China University of Science and Technology, Shanghai 200237, P.R. China; 4 Shanghai Institute of Pharmaceutical Industry, Shanghai 200040, P.R. China

**Keywords:** Bacopaside I, Alzheimer’s disease, β-amyloid, immune, phagocytosis, APP/PS1mice

**This article has been corrected:** Because the authors inadvertently published an unfinished early version of **Figure 3**, errors were present in **Figures 3A** and **3B**. In **Figure 3A**, which presents MALDI-TOF MS/MS results for protein identification, the authors corrected the numbering of the up- and down-regulated proteins in the accordance with the numbering in Table 1. In **Figure 3B**, which shows validating western blots of four differentially expressed proteins, the authors replaced images for GFAP and ATPB with corresponding images from the original data. These alterations do not affect the results or conclusions drawn from this work. The authors would like to apologize for any inconvenience caused.

The new **Figure 3** is presented below.

**Figure 3:**
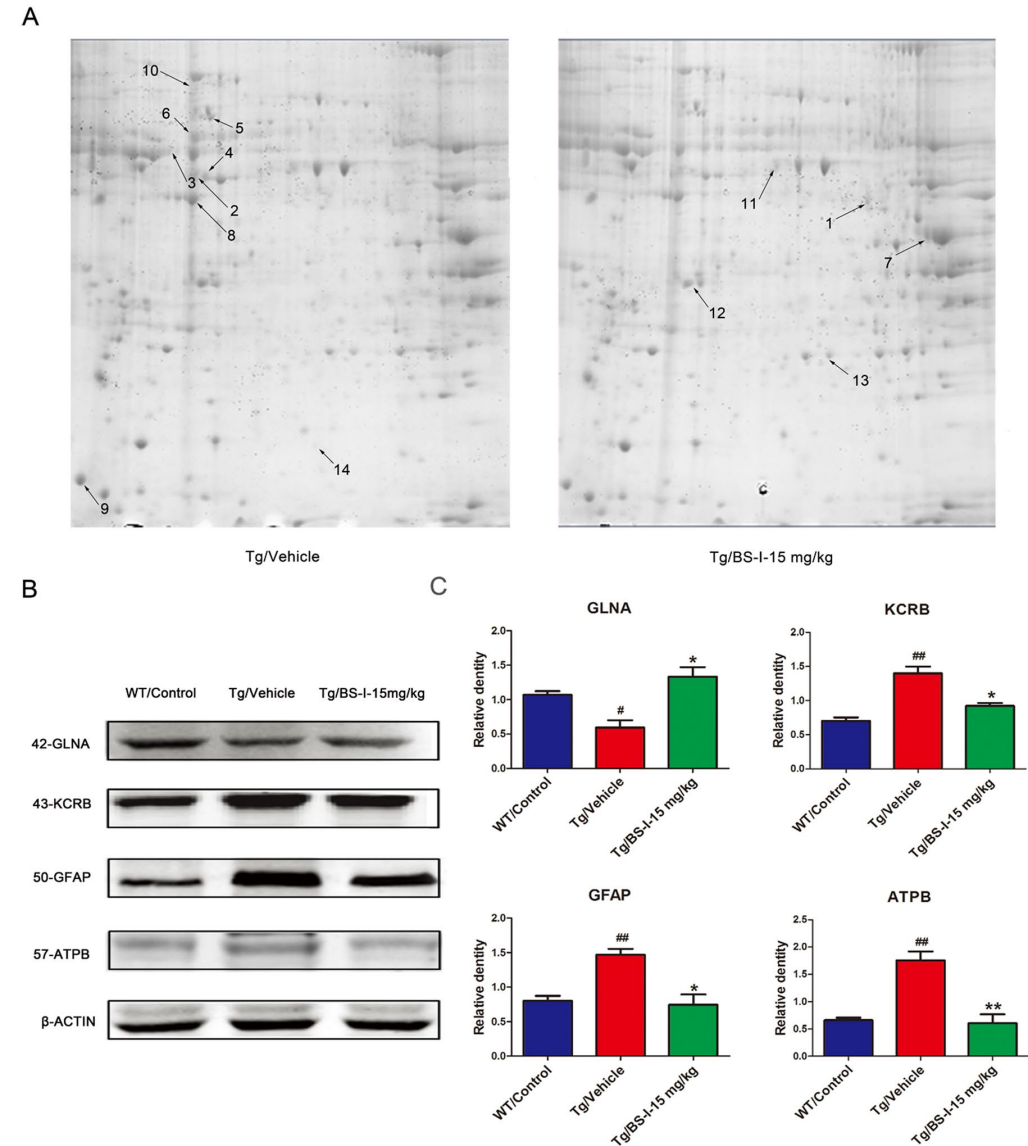
2-DE images and the validation of differentially abundant protein by western blot. The differentially expressed proteins in this study were defined by the gray values of the protein spots. Nine proteins were down-regulated (shown on the Tg/Vehicle gel) and 5 were up-regulated (shown on the Tg/BS-I-15 mg/kg gel) (**A**). To validate the proteomic results, we used a western blot to confirm 4 of the total 14 differentially expressed proteins (**B**). Tg/Vehicle group (M) compared with the wild-type control group (WT), * p < 0.05, ** p < 0.01; Low dose BS-I (15 mg/kg) treated group (L) compared with Tg/Vehicle group, # p < 0.05, ## p < 0.01 (**C**). Error bars denote mean standard error of the mean (SEM), n = 3.

