# Supplementary material for: Bacopaside I ameliorates cognitive impairment in APP/PS1 mice via immune-mediated clearance of β-amyloid
Source: Aging (Albany NY). 2016 Mar 2;8(3):521–33. doi: 10.18632/aging.100913 (PMC4833143; doi:10.18632/aging.100913)
Supplement: Supplementary file 1 [file aging-08-521-s001.pdf]

SUPPLEMENTARY DATA

Novel object recognition test

The NOR test started with a habituation trial during which the animals were placed in the empty open-square gray arena (50*50cm), 35 cm high for 5 min. Three hours later, mice were again placed in the same arena containing two identical objects (familiarization phase). Exploration was recorded in a 10 min trial by an investigator blinded to the genotype and treatment. Twenty-four hours later (test phase), mice were again placed in the arena containing two objects, one of the objects presented during the familiarization phase (familiar object) and a new different one (novel object), and the time spent exploring the two objects was recorded for 10 min. Sniffing, touching, and stretching the head toward the object at a distance of no more than 2 cm were scored as object investigation. Results were expressed as percentage time of investigation on objects per 10 min or as discrimination index (DI), i.e., (seconds spent on novel or seconds spent on familiar)/(total time spent on objects). Animals with no memory impairment spent a longer time investigating the novel object, giving a higher DI.

Bacopaside I treatment improve antioxidant enzyme activities and reduce MDA in mice brain

The results showed that compared to Wild-type control group, 8 months old TG2576 mouse brain antioxidant kinases including superoxide dismutase (SOD), catalase (CAT) and glutathione S-transferase (GSH-Px) activity was significantly lower ($p < 0.01$), SOD enzyme activity values were 150.7 and 116.4, CAT enzyme activity values were 3.0 and 1.8, GSH-Px The enzyme activity values were 199.3 and 68.4 respectively. Bacopaside I treatment (15mg/kg and 50mg/kg), versus vehicle treatment, can increase SOD activity, CAT activity and GSH-Px activity statistically significantly. Besides, Bacopaside I treatment significantly reduced the mice brain lipid metabolic product MDA compared with vehicle treatment ($p<0.05$), indicated that Bacopaside I could significantly inhibit the reaction of lipid metabolism in the brain (a).

SUPPLEMENTARY MATERIALS AND METHODS

Real-time PCR. PCR amplification was performed using the following primers for the indicated genes: GAPHD (5'-CAT CAC CAT CTT CCA GGA GCG-3' and 5'-TGA CCT TGC CCA CAG CCT TG-3'), Psen2A (5'-GCC CGC GGC GTA AAC TCT A-3' and 5'-TGT AGA TGA GCTGCC CGT TC-3'), Psen2B (5'-CTC CGG TTT TGA GCAGTT GTT-3' and 5'-TGA CCT TGC CCA CCT TG-3'), Pik3r2 (5'-ATG CTG AGT GGC AAA CGA GA-3' and 5'-GAG TGT CCA GGCCTG AGT TC-3'), Psen1 (5'-CGC ACT ACT GGA CTG TGG AA-3' and 5'-AAA ACC AGC CAG GACAGA GA-3'), App (5'-GTAGTA GAAGTC GCCGAA GAG G-3' and 5'-CTT TCT GGAAAT GGG CAT GCTCG-3'), Atp2a (5'-TCA GACTGA GAG CAT CCA GG-3' and 5'-TTA TGT TCA GCAGGG TCC AG-3'), Tal1 (5'-TTT CTC CCC GATTCC AAG GC-3' and 5'-AGA CCC ACC AAA AAG GCCAA-3'), Cacna1f (5'-GACTGT GTGCAG ATG GTCCT-3' and 5'-TGT TTG TTG TGC TGG GTCCT-3'), and Prkce (5'-TTA TTG CAT TCC CCC ACC CC-3' and 5'-TTA GGTAGC TGG GCAGGG AT-3').

APP/PS1 mice expressing human APP Swedish mutations (K595N/M596L) and the human presenilin 1 deltaE9 mutation under the regulatory control of the PrP gene promoter on a C57BL/6J back-ground [1] were used to breed a colony of experimental animals.

Kits for the assays of malondialdehyde (MDA), superoxide dismutase (SOD), catalase (CAT) and glutathione peroxidase (GSH-Px) were purchased from Nanjing Jiancheng Bioengineering Institute (Nanjing, People's Republic of China). L-glutamate, dimethyl sulfoxide (DMSO), bovine serum albumin (BSA), Adenosine-5-triphosphate (ATP), Adenosine diphosphate (ADP), Adenosine monophosphate (AMP), 2,3,5-triphenyl tetrazolium chloride (TTC) were all purchased from Sigma-Aldrich Chemical Co. (St. Louis, MO, USA). 2-(2-methoxy-4-nitrophenyl)-3-(4-nitrophenyl)-5-(2, 4-disulfophen-yl)-2H-tetrazolium monosodium salt (WST-8) was purchased from Dojindo, Kumamoto (Japan).

The activity of antioxidant enzymes including superoxide dismutase (SOD), catalase (CAT), glutathione peroxidase (GSH-Px), and malondialdehyde (MDA), was determined 40 min after MWM. Parts of the brain were weighed and homogenized in a buffer consisting of 10 mM sucrose, 10 mM Tris-HCl, and 0.1 mM EDTA (pH 7.4), and subsequently centrifuged at 3000×g for 10 min at 4 °C. The supernatant was used for bioassays. Protein concentrations were measured according to previously described methods [2]to normalize the enzymes activities. The total activity of SOD, expressed as units/mg protein, was determined according to the description in the assay kits. Optical density was determined at 550 nm in a spectrophotometer (UV-2550, Shimadzu, Kyoto, Japan). The formation of stable complex of H_2O_2 with ammonium molybdate was utilized to assay the status of catalase (CAT) activity, and absorption was measured

spectrophotometrically at 405 nm [3]. GSH-Px activity levels in the brain tissues were measured using an assay kit, via the oxidation of NADPH by glutathione reductase [4]. The activity of 1μmol of NADPH oxidation per min, under these conditions, was used as the unit of glutathione reductase activity (expressed as units/mg protein). MDA levels in the brain tissue were detected using assay kit, with a procedure that was slightly modified from previously described methods [5].

SUPPLEMENTARY REFERENCES

1. Dutescu RM, Li QX, Crowston J, Masters CL, Baird PN and Culvenor JG. Amyloid precursor protein processing and retinal pathology in mouse models of Alzheimer's disease. Graefes Arch Clin Exp Ophthalmol. 2009; 247:1213-1221.

2. Lowry OH, Rosebrough NJ, Farr AL and Randall RJ. Protein measurement with the Folin phenol reagent. J Biol Chem. 1951; 193:265-275.

3. Goth L, Meszaros I and Nemeth H. Serum catalase enzyme activity in acute pancreatitis. Clin Chem. 1982; 28:1999-2000.

4. Carlberg I and Mannervik B. Purification and characterization of the flavoenzyme glutathione reductase from rat liver. J Biol Chem. 1975; 250:5475-5480.

5. Rojas P and Rios C. Increased striatal lipid peroxidation after intracerebroventricular MPP+ administration to mice. Pharmacol Toxicol. 1993; 72:364-368.

SUPPLEMENTARY FIGURES

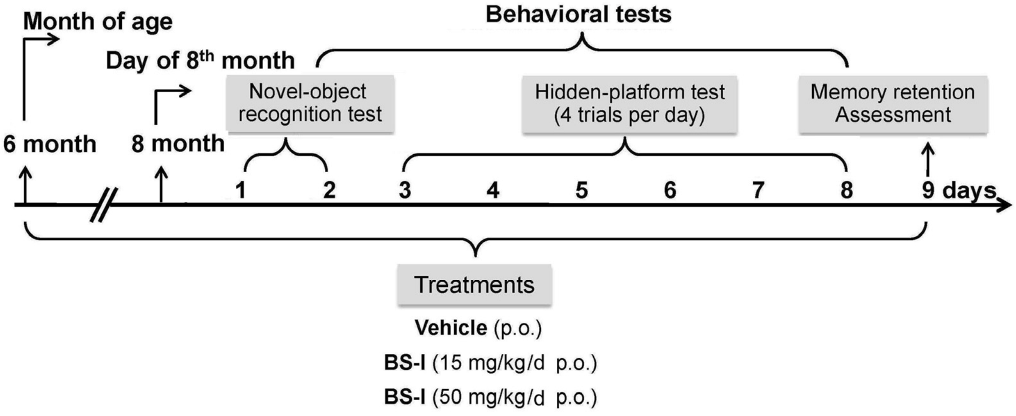

Figure S1. The experimental scheme.

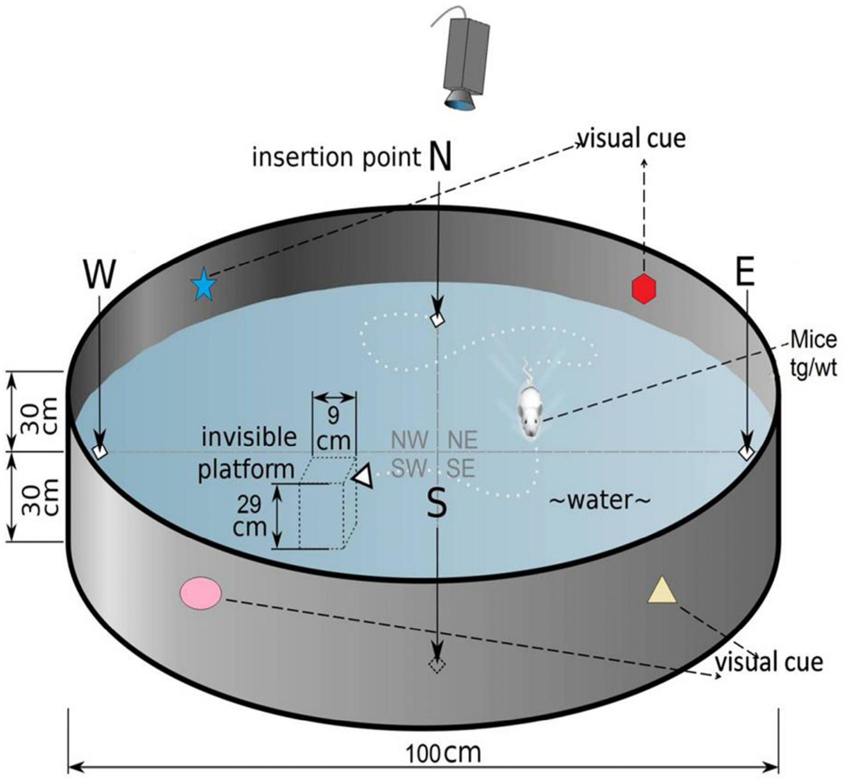

Figure S2. Schematic drawing of the Morris water maze test for mice.

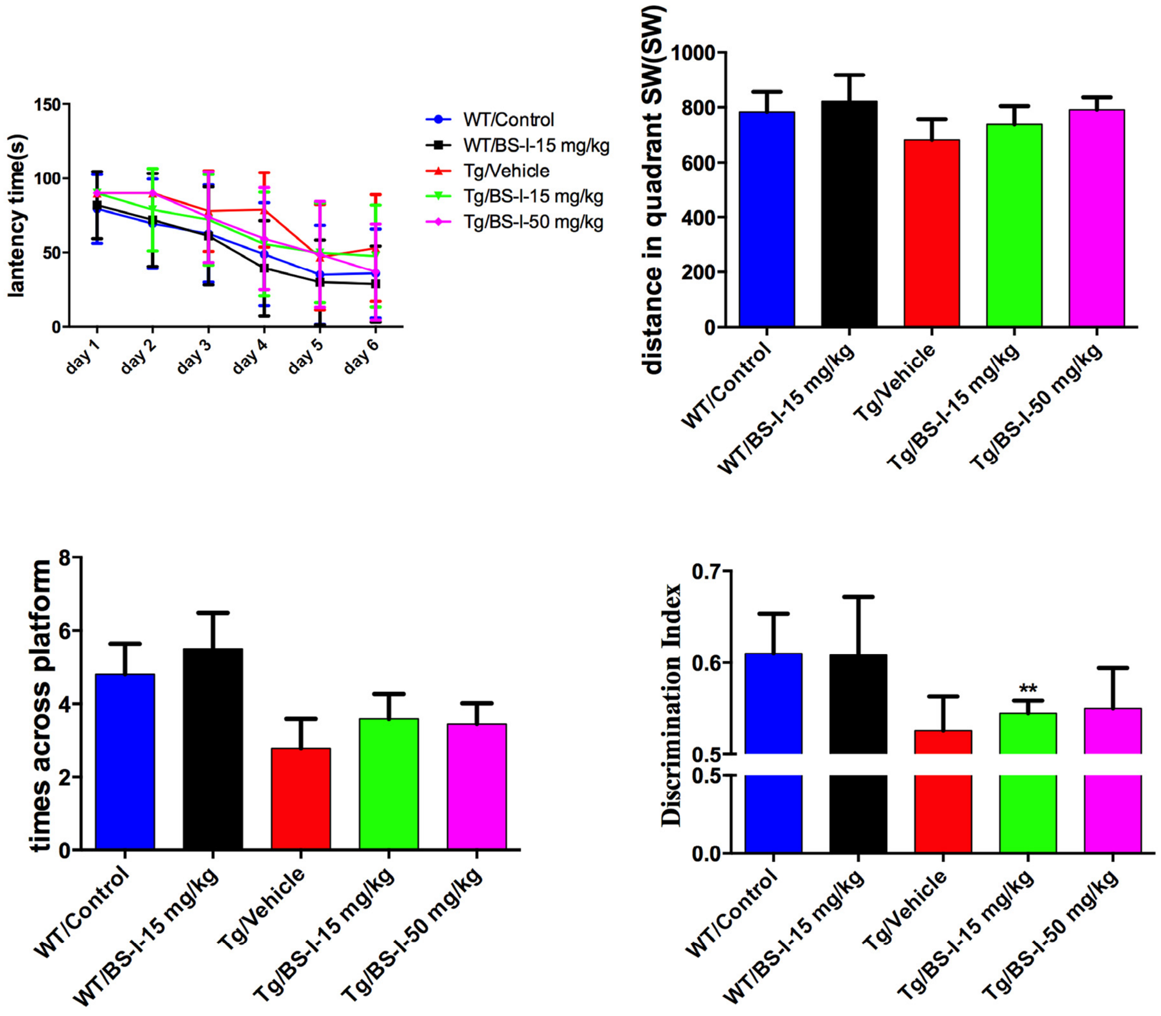

Figure S3. Novel object recognition test and Morris water maze test for the BS-I and vehicle-treated WT and APP/PS1 mice.

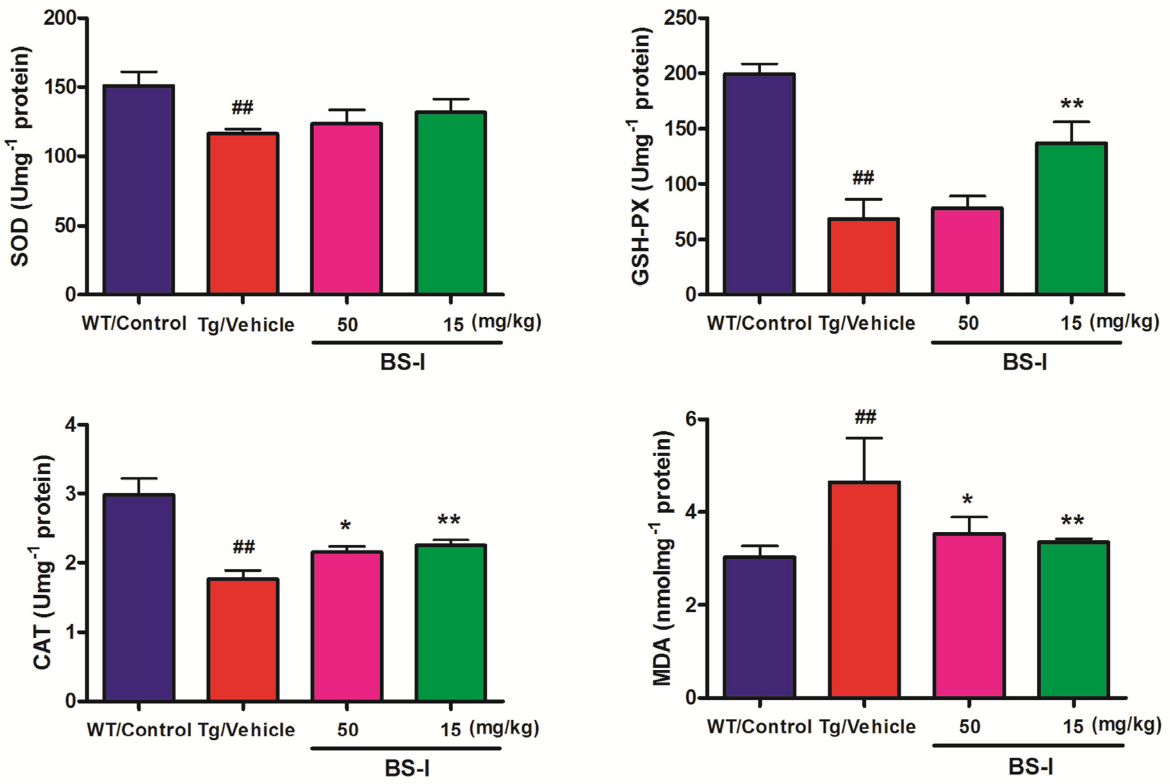

Figure S4. Bacopaside I treated TG2576 mouse brain antioxidant kinase and lipid reaction product of MDA level. TG2576 group compared with the wild-type control group, ## p <0.01; Bacopaside I treated group compared with model group, * p <0.05, ** p <0.01.
